# BioDeformUNet: A Deep Learning Model for Biomechanically Informed Liver Image Registration

**DOI:** 10.64898/2026.08.03.26359612

**Published:** 2026-08-05

**Authors:** Xinyue Zhang, Caleb S. O’Connor, Austin Castelo, McKell E. Woodland, Bilel Daoud, Iwan Paolucci, Jessica Albuquerque, Mais Altaie, Noreen Siddiqi, Ankit B. Patel, Bruno C. Odisio, Kristy K. Brock

## Abstract

**Purpose:**

To build a 3D U-Net model, BioDeformUNet, to predict the liver’s deformation vector field (DVF) in near real-time, for efficient intra-procedural evaluation of the minimal ablative margin (MAM).

**Materials and Methods:**

This retrospective study included 170 contrast-enhanced computed tomography (CECT) image pairs from 157 patients who underwent liver ablation treatment between 2020-2024. Each data instance included one pre-ablation CECT (pre-CECT) and one post-ablation CECT (post-CECT). BioDeformUNet was trained under the guidance of DVFs generated by a biomechanical model-based deformable image registration (DIR) algorithm using a loss function that focused on large liver deformations. Data were split patient-wise into training (92-93 patients), validation (23-24 patients), and testing sets (42 patients). We compared our method’s performance with two deep learning-based DIR methods: VoxelMorph and VFA. Evaluation metrics included: target registration error (TRE), Dice similarity coefficient (DSC), Minimum Ablation Margin (MAM), and inference time. For BioDeformUNet, we additionally evaluated the accuracy of the deformed tumor center-of-mass mapping by comparing the predicted tumor center location with that generated by Morfeus. A mapping error less than 3.0 mm (corresponding to the voxel size) was considered accurate. We used the Wilcoxon signed-rank test to assess the significancy of each test result. Our code is available at https://github.com/XinyueZhang831/BioDeformUNET .

**Results:**

The TRE of BioDeformUNet was not significantly different from Morfeus (3.31±1.17 BioDeformUNet; 3.23±1.06 Morfeus; p-value=0.41). The BioDeformUNet DVF magnitude was within 3.0 mm of Morfeus DVF for an average of 91.9 ± 17.6% (mean ± SD) of the voxels. Tumor mapping errors greater than 3.0 mm occurred in only 8 cases. The inference time of BioDeformUNet was 0.6s per image pair, 0.2s for VoxelMorph, 0.3s for VFA, and 20.2s for Morfeus.

**Conclusion:**

BioDeformUNet achieved a similar performance to the biomechanical model-based algorithm but required fewer computational operations, resulting in a 34× speedup in DVF computation.

## Full Text Availability

The license terms selected by the author(s) for this preprint version do not permit archiving in PMC. The full text is available from the preprint server.

